# Supplementary material for: Finite-time generalized synchronization of non-identical fractional order chaotic systems and its application in speech secure communication
Source: PLoS One. 2022 Mar 23;17(3):e0263007. doi: 10.1371/journal.pone.0263007 (PMC8942225; doi:10.1371/journal.pone.0263007)
Supplement: Attachment — Submitted filename: PONE-D-21-24308 comments.pdf [file pone.0263007.s002.pdf]

Comments PONE-D-21-24308:

This paper provide some solutions of finite-time generalized synchronization problem for the nonidentical fractional order chaotic (or hyper-chaotic) systems via designing adaptive sliding mode controller. In my opinion, the article is innovative and suitable for publication in this journal after the following revisions can be finished.

1 This article needs further careful examination to avoid language errors that should not occur, for example at the beginning of the **Introduction**, there are two Chaos Chaos; Title of Chapter II： “**2. Basic definition and lemma**” I think “**definitions**” should be more suitable.

2. The reference format of the article is very chaotic, and the author needs to revise it uniformly according to the requirements of the journal.

3. The relationship between theorem 1 and theorem 2 is not well explained, which is easy to cause confusion in understanding. Please explain in detail the relationship between theorem 1 and theorem 2.

4. Figure 1-4 only list the behavior evolution of system state and output, without further detailed comparative analysis. Please give a detailed comparative analysis of status and output signals.
