# Supplementary material for: Finite-time generalized synchronization of non-identical fractional order chaotic systems and its application in speech secure communication
Source: PLoS One. 2022 Mar 23;17(3):e0263007. doi: 10.1371/journal.pone.0263007 (PMC8942225; doi:10.1371/journal.pone.0263007)
Supplement: Attachment — Submitted filename: Comments to PONE-D-21-24308R1.pdf [file pone.0263007.s004.pdf]

Comments to PONE-D-21-24308R1

Authors have made good modifications and basically answered my questions. I think it's acceptable.
