# Supplementary material for: Finite-time generalized synchronization of non-identical fractional order chaotic systems and its application in speech secure communication
Source: PLoS One. 2022 Mar 23;17(3):e0263007. doi: 10.1371/journal.pone.0263007 (PMC8942225; doi:10.1371/journal.pone.0263007)
Supplement: Attachment — Submitted filename: Response to Reviewers_11.26.docx [file pone.0263007.s003.docx]

**Original Manuscript ID:** PONE-D-21-24308

**Original Article Title: “**Finite-time generalized synchronization of non-identical fractional order chaotic systems and its application in speech secure communication”

**To:** PLOS ONE Editor

**Re:** Response to reviewers

Dear Editor,

Thank you for allowing a resubmission of our manuscript, with an opportunity to address the reviewers’ comments.

We thank the innominate reviewers for their valuable comments and suggestions in improving the quality of the manuscript. We give the following replies in the order of reviewers.

We are uploading (a) our point-by-point response to the comments (below) (response to reviewers), (b) an updated manuscript with yellow highlighting indicating changes, and (c) a clean updated manuscript without highlights (PDF main document).

Best regards,

Jianxiang Yang

**Comments from Reviewer 1**

We have modified the new manuscript following your advice. Thank you for your suggestion again. Main contents are listed as follows.

**Reviewer#1, Concern # 1:** **This article needs further careful examination to avoid language errors that should not occur, for example at the beginning of the Introduction, there are two Chaos Chaos; Title of Chapter II：“2. Basic definition and lemma”I think “definitions”should be more suitable.**

**Author response:** We have modified the new manuscript following your advice. Thank you for your suggestion again.

**Author action:** We updated the manuscript with red highlighting in the introduction and Title of Chapter II.

**Reviewer#1, Concern # 2:** **The reference format of the article is very chaotic, and the author needs to revise it uniformly according to the requirements of the journal.**

**Author response:** We add some latest references according to the requirements of the journal in the new submission.

**Author action:** We updated the manuscript in reference section.

**Reviewer#1, Concern # 3:** **The relationship between theorem 1 and theorem 2 is not well explained, which is easy to cause confusion in understanding. Please explain in detail the relationship between theorem 1 and theorem 2.**

**Author response:** We provide a more detailed discussion in the new submission. Main contents are listed as follows.

**Remark 5.** Theorem 1 has proved that the synchronization error (11) can be achieved to be zero within a finite time, and Theorem 2 has proved that the state trajectories of the error system can converge to zero within a given finite time. Therefore, according to Theorems 1 and 2, the upper bound of total convergence time can be estimated as$T^{*}<{T_{1}^{*}+T}_{2}^{*}$.

**Author action:** We updated the manuscript by with red highlighting in **Remark 5**.

**Reviewer#1, Concern # 4:** **Figure 1-4 only list the behavior evolution of system state and output, without further detailed comparative analysis. Please give a detailed comparative analysis of status and output signals.**

**Author response:** Figure 1and Figure 3 show the phase portraits and time response of master system respectively, and Figure 2and Figure 4 show the phase portraits and time response of slave system respectively. The detailed comparative analysis and explanation are demonstrated by using two varieties of cases for non-commensurate order and commensurate order as shown in Figs. 6 and 9.

**Author action:** We updated the manuscript by with red highlighting throughout this paper.

**Comments from Reviewer 2**

We have modified the new manuscript following your advice. Thank you for your suggestion again. Main contents are listed as follows.

**Reviewer#2, Concern # 1:** **What are the advantages of the fractional order system considered in this paper over the integer order system? What are the differences and difficulties in handling?**

**Author response:** The main reason for using the integer-order models was the absence of solution methods for fractional differential equations. The advantages or the real objects of the fractional order systems are that we have more degrees of freedom in the model and that a ‘memory’ is included in the model. And it has been shown that the fractional calculus can be applied as an important mathematical tool for exact modeling of practical systems.

Furthermore, we proposed finite-time generalized synchronization method is fully suitable for synchronizing all types of instances in fractional order dynamical systems, and the rate of synchronization error of proposed robust synchronization is very less (approximately 1 second) than the existing sliding mode synchronization without uncertainties and external disturbances. Note that, in secure communication process, the synchronized systems with minimum synchronization error are produced higher security and efficient results. A new audio cryptosystem is introduced for sharing voice messages via insecure channel secretly based on the proposed synchronization technique. Likewise, the security of the proposed cryptosystem is analyzed. Additionally, the stability conditions for achieving the generalized synchronization are explicitly derived in finite time.

**Author action:** We updated the manuscript in the introduction.

**Reviewer#2, Concern # 2:** **The contributions of this paper need to be improved.**

**Author response:** Inspired by aforementioned previous works, the contributions of this paper can be summarized on four aspects.

(1) Based on the definition of the generalized synchronization, the synchronization schemes of two non-identical fractional order chaotic systems are proposed to achieve finite-time generalized synchronization with considering the uncertainties and external disturbances.

(2) To study generalized synchronization, a novel fractional order integral sliding mode surface is designed and its stability to the origin is proved in a given finite time.

(3) According to the fractional Lyapunov stability theory, an appropriate sliding mode controller with adaptive update laws is proposed under external disturbances and model uncertainties, and the stability conditions for achieving the generalized synchronization are explicitly derived in finite time.

(4) Numerical simulation results further highlight the validity, the novelty and applicability of the proposed approach for non-identical fractional order chaotic systems. For the application of secure communication process, a new speech encryption system is introduced to share voice messages secretly via secure channel in terms of the proposed synchronization criterion. Meanwhile, the security of the proposed theories is also analyzed and discussed.

**Author action:** We updated the manuscript in the introduction.

**Reviewer#2, Concern # 3:** **The background in the introduction part is not enough, please refer to the following reference:
IEEE Transactions on Neural Networks and Learning Systems, DOI: 10.1109/TNNLS.2019.2952410.**

**Author response:** In the introduction of this new manuscript, we add some latest references to obtain a more comprehensive review of literature. Please see the reference [23].

**Author action:** We updated the manuscript in the introduction.

**Reviewer#2, Concern # 4:** **In the Definitions 1-2, Lemmas 2, 3, 5 in the paper, they require \alpha \in (0,1), while Lemma 1 requires \alpha \in (0,1], which makes the definitions 1-2, Lemmas 2, 3, 5 no longer hold when Lemma 1 holds. Please explain this contradiction issue in detail.**

**Author response:** Lemma 1 has been revised in the new manuscript as follows：

**Lemma 1** When the fractional-order derivative${{}_{t_{0}}^{C}D}_{t}^{\alpha}x\left( t \right)$is integrable, let $\Omega=\left[ a,b \right]$ be an interval on the real axis$R$, and let $n=\left[ \alpha\right]+1$for $\alpha\notin N$or $n=\alpha$for $\alpha\in N$. If $x(t)\in C^{n}\left[ a,b \right]$, one can obtain:

${{}_{a}^{C}I}_{t}^{\alpha}{{}_{a}^{C}D}_{t}^{\alpha}x\left( t \right)=x\left( t \right)-\sum_{k=0}^{n-1} \frac{\left. x^{\left( k \right)}(a \right)}{k!}\left( t-a \right)^{k},n-1<\alpha\leq n$	(3)

Especially, if $0<\alpha\leq1$ and $x(t)\in C^{1}\left[ a,b \right]$, then ${{}_{a}^{C}I}_{t}^{\alpha}{{}_{a}^{C}D}_{t}^{\alpha}x\left( t \right)=x(t)-x(\left. a \right).$

In other words, when$\alpha=1$, it becomes an integer order equation$\int_{a}^{b} \dot{x}(t)dt=x(b)-x(\left. a \right)$.

**Author action:** We updated the manuscript with bold black indicating changes.

**Reviewer#2, Concern # 5:** **The symbol “\varepsilon_{i}” given in Lemma 4 is not reflected in the following inequality (6), and the symbol \xi in (6) does not give a corresponding definition. In addition, there are many symbols in the full paper that appear for the first time without giving corresponding definitions, such as the symbol \tau, the symbol before the function f(t) in definition 1, etc.**

**Author response:** There was a typing mistake **in Lemma 4,** and it was updated as follow:

**Lemma 4** If $d_{i}\mathbb{\in R,}i=1,2\cdots n$ and $\xi\in\left( 0,1 \right)$ are arbitrary real numbers, the following inequalities satisfy:

${|d_{1}|}^{\xi}+{|d_{2}|}^{\xi}+\cdots+{|d_{n}|}^{\xi}\geq\left( |d_{1}|+|d_{2}|+\cdots+|d_{n}| \right)^{\xi}$	(6)

Other symbols in the full paper were given corresponding definitions, and some errors have been proofread carefully in the new submission.

**Author action:** We updated the manuscript by with red highlighting in definitions section.

**Reviewer#2, Concern # 6:** **The authors mentioned in Remark 4 that “designing an appropriate control law ′() for any different dimensional systems (9) and (10)”? Is this designed control law applicable to any different dimensional systems?**

**Author response:** Defining$\left. e(t)=\varphi(y)-\phi(x \right)$, from system (9) and system (10), in order to introduce conveniently, the control signals$U\left( t \right)$ are considered as$U^{'}\left( t \right)$and$U^{''}\left( t \right)$, i.e., $U\left( t \right)=U^{'}\left( t \right)+U^{''}\left( t \right)$.The compensation controller$U^{''}\left( t \right)$ can be preset as$U^{''}\left( t \right)=J_{\varphi}^{-1}J_{\phi}\left( D^{\beta}x-D^{\alpha}x \right)$, and the separated controller $U^{'}\left( t \right)$will be designed later. Therefore, for any different dimensional systems (9) and (10) with disturbances and model uncertainties, designing an appropriate control law$U^{'}\left( t \right)$ can achieve generalized synchronization.

**Author action:** We updated the manuscript with bold black indicating changes.

**Reviewer#2, Concern # 7:** **What is the prior knowledge of the upper bound of the system mentioned by the authors in Remark 6? In the control scheme proposed in this paper, why is it not necessary to give a priori knowledge of the upper bound? What method was used to deal with it?**

**Author response:** In Remark 6, the prior knowledge of the upper bounds refers to the knowledge of the system model or the dynamical system upper bound, our proposed control scheme does not know this information. And the control input is designed as $U\left( t \right)=U^{'}\left( t \right)+U^{''}\left( t \right)$. The compensation controller$U^{''}\left( t \right)$ can be preset as$U^{''}\left( t \right)=J_{\varphi}^{-1}J_{\phi}\left( D^{\beta}x-D^{\alpha}x \right)$, the adaptive sliding controller ${U^{'}}_{i}\left( t \right)$ is proposed as shown in Eq. (24). The adaptive update laws are listed in Eq. (25), and the disturbances and model uncertainties need to satisfy the Assumption 1 and 2. Further, the control inputs ${U^{'}}_{i}\left( t \right)$ are feasible in practical applications.

**Author action:** We updated the manuscript with bold black indicating changes.

**Reviewer#2, Concern # 8:** **The expression of the finite time T calculated in the paper contains the initial values of some variables. How are these initial values determined?**

**Author response:** The initial conditions of $\hat{\varepsilon}_{i}\left( 0 \right)\left( i=1,2,3 \right)$and$\hat{\gamma}_{i}\left( 0 \right)\left( i=1,2,3 \right)$ are chosen as 0.5, the control parameters are chosen as$k_{1}=k_{2}=10$, both *δ* and *σ* are set equal to 0.8. [It's](javascript:;) noted [that](javascript:;) the controller is performed at$t_{0}=20s$and start from initial state$\left. \left( \left. x_{1}(20),x_{2}(20),x_{3}(20 \right),x_{4}(20) \right)=(8.38,5.89,12.08,89.39 \right), \left. \left( \left. y_{1}(20),y_{2}(20),y_{3}(20 \right) \right)=(2.43,0.75,-2.83 \right)$, thus.

According to Theorem 1, the states of the sliding mode dynamics system (14) will converge within a given time

$$T_{1}^{*}=t_{0}+\left( \left\| e\left( t_{0} \right) \right\|_{1}^{\beta-\delta}\frac{\Gamma\left( 1-\delta\right)\Gamma\left( 1+\beta\right)}{k_{1}\Gamma\left( 1+\beta-\delta\right)} \right)^{\frac{1}{\beta}}$$

$$={0+\left( \left\| \left. e(20 \right) \right\|_{1}^{0.99-0.8} \right)}^{\frac{1}{0.99}}\times\left( \frac{\Gamma\left( 1-0.8 \right)\Gamma\left( 1.99 \right)}{10\times\Gamma\left( 1.99-0.8 \right)} \right)^{\frac{1}{0.99}}=1.2298$$

Then, based on Definition 1 and Theorem 2, the estimated time is calculated as

$$T_{2}^{*}=t_{0}+\left( \begin{aligned} \left( \begin{aligned} \frac{1}{2}s_{i}^{2}\left( 0 \right)+\frac{1}{2}\left( \begin{aligned} \hat{\varepsilon}_{i}\left( 0 \right) \\ -\varepsilon_{i} \end{aligned} \right)^{2}+\frac{1}{2}\left( \begin{aligned} \hat{\gamma}_{i}\left( 0 \right) \\ -\gamma_{i} \end{aligned} \right)^{2} \end{aligned} \right)^{\beta-\frac{\sigma+1}{2}}\frac{\Gamma\left( 1-\frac{\sigma+1}{2} \right)\Gamma\left( 1+\beta\right)}{2^{\frac{\sigma+1}{2}}k_{2}\Gamma\left( 1+\beta-\frac{\sigma+1}{2} \right)} \end{aligned} \right)=1.222$$

**Author action:** We updated the manuscript in numerical simulation section.
